# Parental preferences for the procedural sedation of children in dentistry: a discrete choice experiment

**DOI:** 10.3389/fped.2023.1132413

**Published:** 2023-12-05

**Authors:** Jinru Zhuge, Dongyue Zheng, Xingwang Li, Xin Nie, Jiefan Liu, Ruohai Liu

**Affiliations:** ^1^ Department of Anesthesiology, The Affiliated Stomatology Hospital of Wenzhou Medical University, Wenzhou, China; ^2^ Department of Nursing, The Second Affiliated Hospital of Wenzhou Medical University, Wenzhou, China; ^3^ Department of Anesthesiology, The Second Affiliated Hospital of Wenzhou Medical University, Wenzhou, China; ^4^ Department of Stomatology, The Affiliated Stomatology Hospital of Wenzhou Medical University, Wenzhou, China

**Keywords:** discrete choice experiment, preference, procedural sedation, pediatric dentistry, dental anxiety

## Abstract

**Purpose:**

The aim of this study was to explore parental preferences for the procedural sedation of children in dentistry through a discrete choice experiment (DCE) to inform clinical decisions and oral health management.

**Methods:**

Based on literature reviews, interviews with parents of pediatric dental patients, and expert consultation, six attributes, including fasting time, recovery time, sedative administration routes, adverse reactions, sedation depth and procedure cost, were incorporated into the DCE questionnaire. The DCE questionnaire collected data on parental preferences for pediatric dental sedation treatment from June to August 2022. A conditional logit model was used to analyze preference and willingness to pay (WTP) for each attribute and its level. Subgroup analyses assessing the impact of parents' dental anxiety on procedural sedation preferences were also conducted using conditional logit models.

**Results:**

A total of 186 valid questionnaires were gathered. Parents' preferences for fewer adverse reactions, a milder sedation depth, lower out-of-pocket cost, shorter fasting and recovery times and administration by inhalation were significantly associated with their choice of sedation model. The conditional logit model showed that parents were most interested in treatments with no adverse reactions (0% vs. 15%) (Coef, 1.033; 95% CI, 0.833–1.233), followed by those providing minimal sedation (vs. deep sedation) (Coef, 0.609; 95% CI, 0.448–0.769). Moreover, the relative importance of adverse reactions and fasting time was higher among anxious than nonanxious parents. The study found a WTP threshold of ¥1,538 for reducing adverse reactions (15% to 0%). The WTP threshold for the best sedation procedure scenario (no fasting requirement, 10 min recovery time, administration by inhalation, 0% adverse reaction incidence and minimal sedation) was ¥3,830.

**Conclusion:**

Reducing the adverse reactions and depth of sedation are predominant considerations for parents regarding procedural sedation in pediatric dentistry, followed by lower cost, shorter fasting and recovery times and inhalation sedation. Parents with dental anxiety had a stronger preference for options with a lower incidence of adverse reactions and shorter fasting time than parents without dental anxiety. This discovery is helpful for doctors and can promote collaborative decision-making among parents and doctors.

## Highlights

• For the procedural sedation of children in dentistry, parents were most interested in options with fewer adverse reactions and a milder depth of sedation.
• Parents with dental anxiety had a stronger preference for options with a lower incidence of adverse reactions and shorter fasting time than parents without dental anxiety.
• The willingness to pay (WTP) threshold for the best sedation scenario (no fasting requirement, 10 min recovery time, administered by inhalation, no adverse reaction and minimal sedation) was ¥3,830.

## 1. Introduction

Children often have insufficient coping skills for dental treatments, making it difficult to provide quality dental care to children. Dental diseases can cause pain, sleep disruption, difficulty acquiring knowledge, and poor growth in children, while uncomfortable dental treatment experiences can cause psychological harm (1, 2). Children's dental anxiety is widespread, with a prevalence of 5%–20% among children and adolescents (3). Importantly, parental anxiety has been identified as a factor to children's dental anxiety, as anxious parents may inadvertently transmit their own fears and apprehensions to their children, further exacerbating the child's anxiety during dental visits (4). Children's dental anxiety is derived from dental diseases and poor oral health, such as untreated dental infections and decay, which negatively affect an individual's quality of life (5, 6). Moreover, dental anxiety in childhood can lead to dental anxiety and fears in adulthood, which has long-lasting effects on health later in life (7, 8). Studies have revealed that approximately 10%–20% of the population avoids necessary dental treatments due to anxiety, thus missing out on optimal interventions, reducing quality of life and causing a significant financial burden on families and society (9, 10). Therefore, dental anxiety in children, and the influence of parental anxiety, have long been acknowledged as sources of problems in patient management (4, 11).

Procedural sedation as a pharmacological means of behavior management is used in dentistry to relieve dental anxiety and has been widely applied to children (12). Deep sedation is often used as an alternative to general anesthesia for invasive pediatric dental procedures and has proven safer and cost-effective (13). Studies have revealed that parents' attitudes to pediatric dental care directly correlate with their children's dental health (14). At present, the procedural sedation of children yields a generally high level of satisfaction in parents due to reduced dental anxiety and smoother dental procedures (15). The procedural sedation of children in dentistry is conducted only after parents have been informed and given their consent (16).Therefore, even if the dentist believes that the outcome of pediatric dental care performed under sedation is mostly satisfactory, understanding parental preferences for the procedural sedation of children in dentistry is essential.

Discrete-choice experiments (DCEs) are quantitative methods for measuring the strength of an individual's preferences (17). In healthcare and health economics, DCEs are increasingly advocated for in benefit-risk trade-off assessments for medications, health technologies, and health assessment services to facilitate public decision-making, and related research is increasing yearly (18–20). DCEs have also been applied to study the preferences and willingness to pay of patients and doctors in dentistry (21, 22). In dental procedural sedation, one DCE-based study revealed that the incidence of adverse events, parental concerns, and physician practice times all influence physician choice regarding the fasting time required for the dental procedural sedation of children (23). However, there is little evidence relating to parental preferences for attributes of procedural sedation options in pediatric dentistry and the relative importance they place on the different characteristics that describe a sedation procedure.

Children are a large and unique group, and improving access to quality pediatric dental care is an important medical, public health and social issue. Procedural sedation is currently a common and effective way of improving pediatric dental care quality. The primary objective of this study is to investigate parental preferences for various attributes of procedural sedation in pediatric dentistry using a DCE approach. The secondary objective is to assess the influence of parental anxiety on their preferences for different sedation attributes. The hypothesis of the project is that parents consider safety attributes of sedation to be more important than other factors when consenting to pediatric procedural sedation in dentistry.

## 2. Methods

### 2.1. Identification of attributes and levels

In this study, we used a DCE survey methodology to assess the procedural sedation preferences of parents in pediatric dentistry in China. DCEs are increasingly used in healthcare settings to examine patients' and their families’ preferences in hypothetical alternative scenarios (24–27). Two or more constructed therapeutic options comprising different attributes and attribute levels are presented, requiring choice experiment respondents to choose their favorite options; individual differences in attributes influencing decision-making are then calculated (19, 28).

Identifying attributes and their levels is the most important step to ensure the validity of the DCE (29). In this study, the attributes and their levels were specified using literature reviews (15, 23, 30–36), interviews with parents of pediatric dental patients, and expert consultation. Thus, the DCE used in our study included 6 attributes recorded at 3 or 4 levels. Specified attributes were related to fasting time, recovery time, the administration route of sedatives, adverse reactions, the depth of sedation and the cost of procedural sedation (Table 1). In the Supplementary material Table S1 presents a list of all initial attributes considered during the qualitative research phase.

**Table 1:** The attributes and levels included in the DCE.

Attributes	Levels
Fasting time The period before treatment during which eating and drinking is not permitted	No requirement
	0 h for clear fluids
	3 h for milk and solids
	2 h for clear fluids
	6 h for milk and solids
Recovery time The time from the end of the procedure to time the patient returns to baseline status and is allowed to leave the hospital	10 min
	30 min
	90 min
Administration of sedatives The administration route of the sedatives used for procedural sedation per visit	Inhalation sedation
	Oral sedation
	Intravenous sedation
	Intranasal sedation
Adverse reaction incidence Common adverse reactions are nausea and vomiting, respiratory (respiratory rate and tidal volume), circulatory depression (heart rate and blood pressure drop) and aspiration	0 in 100 people (0%)
	5 in 100 people (5%)
	15 in 100 people (15%)
Sedation depth The depth of sedation produced per visit	Minimal sedation (awake and calm)
	Moderate sedation (sleepy)
	Deep sedation (asleep and cannot be easily roused)
Cost The cost of this procedural sedation per visit	¥100
	¥200
	¥400
	¥800

### 2.2. Construction of the DCE questionnaire

**Figure 1:**
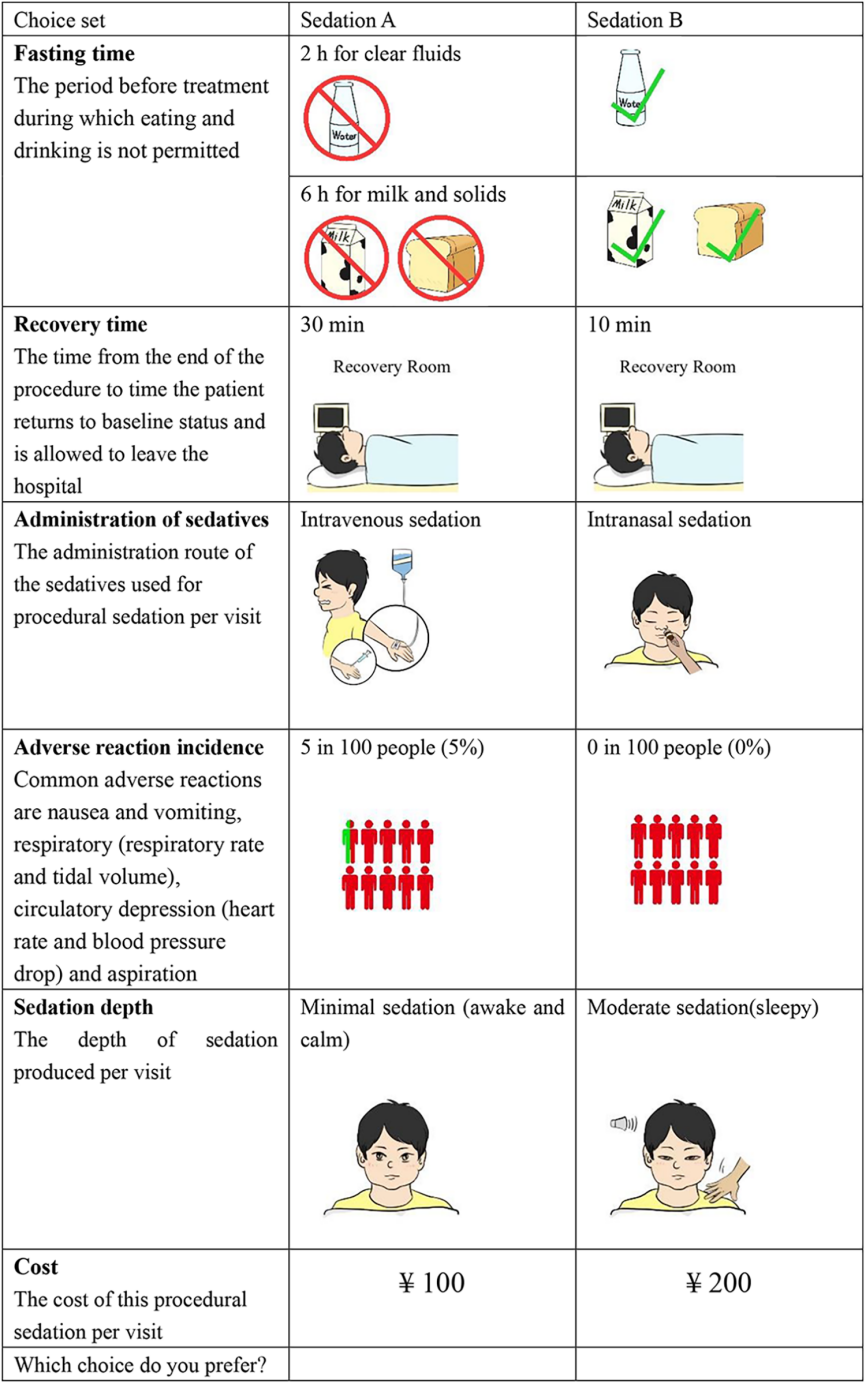
Example of the choice set in the questionnaire.

The study obtained 1,296 (3 × 3 × 4 × 3 × 3 × 4) hypothetical scenarios by combining attributes and levels (six attributes with three or four levels per attribute). It is not realistic for the patient to complete all the choice sets. Therefore, the D-optimal design (SAS 9.4, the procedure by %ChoicEff Macro) was used to generate the best scenarios (37). The experimental design consisted of 24 choice sets, all of which were binary choices (an example of a choice set is shown in Figure 1). These 24 choice sets were further divided into 2 questionnaire versions to minimize the burden on the respondents, and each respondent was required to complete 12 trade-off questions. An additional repeated choice task was added to the questionnaire to check the consistency of responses.

In May 2022, a pilot survey was conducted among 10 parents of pediatric dental patients to improve the phrasing, formatting and question layout by checking their understanding of the wording and the time taken to complete the DCE.

### 2.3. Participants

The study was authorized by the ethics committee of the Hospital of Stomatology, Wenzhou Medical University (WYKQ2022006), and all participants provided written informed consent to participate.

In this study, we continuously recruited the parents of children preparing to undergo dental treatments in the children's dental department of the Hospital of Stomatology, Wenzhou Medical University, China. The inclusion criteria were as follows: (1) these children would receive dental treatments; (2) these children aged less than 12 years old and older than 2 years old; (3) no cognitive impairment and understanding of the questionnaire; and (4) willing to participate and provide informed consent. The exclusion criteria were as follows: (1) incomplete questionnaire content and (2) wrong answers to the consistency check. The parents were interviewed face to face with trained interviewers, which might help respondents understand the questionnaire.

From 1 June 2022 to 5 August 2022, 200 patients consented to complete the survey. Among the participants, 14 were excluded because they did not correctly respond to the consistency check, indicating that they did not fully comprehend the task. The final sample in our study included 186 respondents. The Orme equation revealed that this number was large enough for reliable statistical analyses, as per a related study (38).

In addition to DCE preferences, the survey collected information about children's and parents' sociodemographic characteristics. Parents' dental anxiety was measured by the Visual Analog Scale for Anxiety (VAS-A), an efficacious, fast and manageable tool for screening anxiety states; VAS-A values ≥50 mm indicated a degree of dental anxiety (39).

### 2.4. Statistical analysis

Analytical Statistics Clinical parameters and sociodemographic traits were assessed using descriptive analyses. The conditional logit model was used to analyze the DCE data using STATA statistical software (version 15) (40). All attribute levels were dummy-coded except the cost of procedural sedation, which was a continuous variable. The model provides coefficients representing the effect of each attribute level on predicting individual preferences and their statistical significance. A higher coefficient indicates that the related attribute level is of greater importance for participants considering the choice. Subgroup analyses assessing the impact of parents' dental anxiety on the preferences for procedure sedation were also conducted using conditional logit models, and the relative importance of each attribute in decision-making was calculated. The willingness to pay (WTP) threshold was defined as the amount of money representing an individual's marginal payments for the altered attribute levels in a new alternative scenario (41). Physicians can use the WTP to assess and compare the economic value of each attribute. The WTP estimates were calculated by the nlcom procedure in STATA 15. *p-*values were 2-sided, and the level of statistical significance was set at *p* < 0.05.

## 3. Results

### 3.1. Characteristics of the sample

The final sample comprised 186 parents of pediatric dental patients. The demographics and characteristics of the respondents are shown in Table 2. The parents' ages ranged from 26 to 49 years, with an average of 36 years, and the majority were female (64.0%) and married (97.8%). Most respondents (86.0%) had a tertiary education degree or above, and 55.9% had an annual family income over 150 thousand yuan (approximately 21 thousand US dollars). According to the VAS-A results, 55.9% of parents had a degree of dental anxiety regarding their children. The children were 55.4% male, with an average age of 7.3 years. Most of the children had received previous dental treatment (77.4%).

**Table 2:** Characteristics of the study sample.

Variable	Parents (*N* = 186)
Sex, *n* (%)	
Male	67 (36.0)
Female	119 (64.0)
Age (years), mean, [range]	36.0, [26–49]
Marital status, *n* (%)	
Married	182 (97.8)
Unmarried	0 (0)
Divorced	4 (2.2)
Educational level, *n* (%)	
Low (≤6 years)	2 (1.1)
Medium (6–≤9 years)	24 (12.9)
High (>9 years)	160 (86.0)
Annual household income (yuan), *n* (%)	
<50 k	12 (6.5)
50 k–150 k	70 (37.6)
>150 k	104 (55.9)
Anxiety score (VAS-A), *n* (%)	
<50 mm	82 (44.1)
≥50 mm	104 (55.9)
Sex of the children, *n* (%)	
Male	103 (55.4)
Female	83 (44.6)
Age (years) of the children, mean, [range]	7.3, [2–12]
Dental procedure history of the children	
Yes	144 (77.4)
No	42 (22.6)

### 3.2. Parental preference for procedural sedation

**Figure 2:**
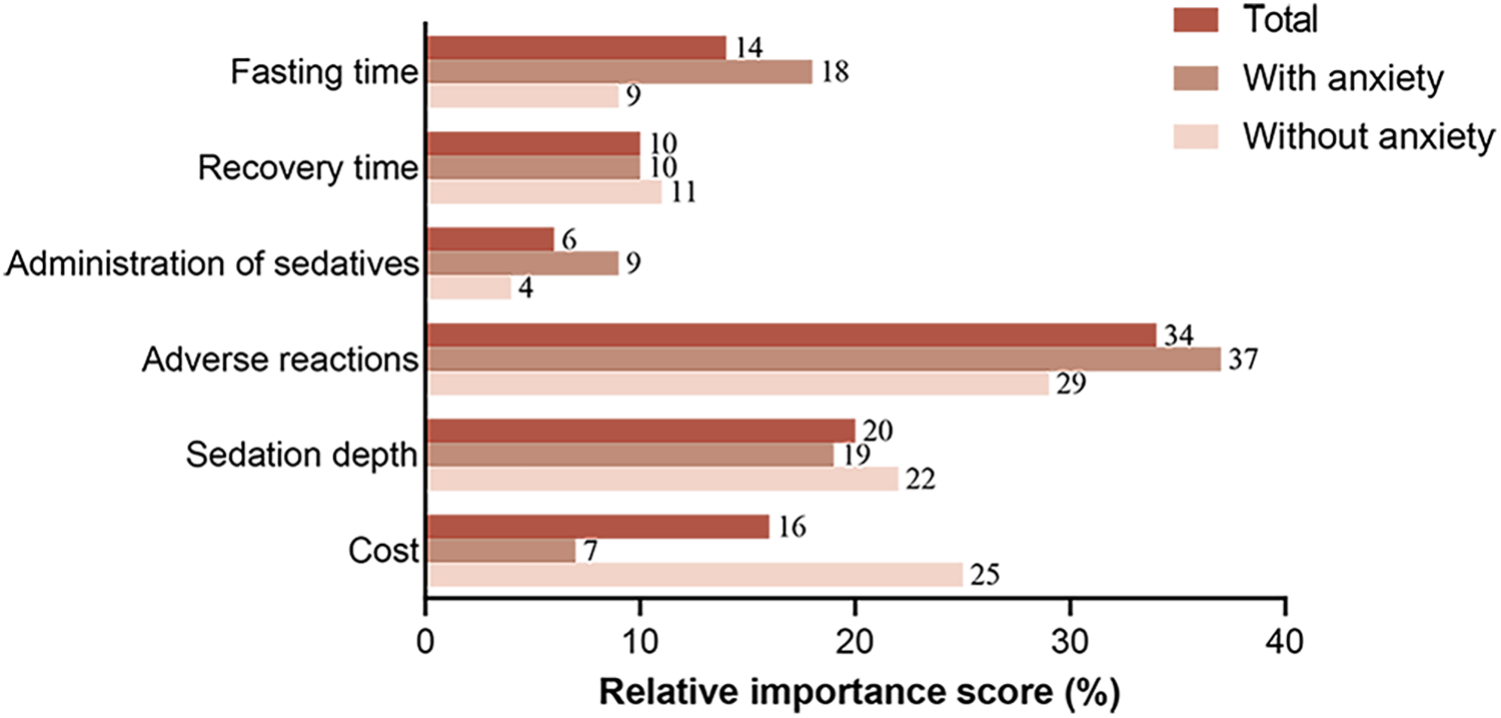
Relative importance score for procedural sedation in parents with and without dental anxiety.

The results of the conditional logit model are shown in Table 3. All covariates were significant except the administration route of the sedative (vs. intravenous sedation) [oral: Coef, 0.069 (95% CI, −0.091–0.230); intranasal sedation: Coef, −0.037 (95% CI, −0.181–0.107)]. Based on the relative importance score in the conditional logit model, adverse reactions and depth of sedation were the most important attributes to parents (34% and 20%, Figure 2). Moreover, the parents were most interested in options with no adverse reactions (0% vs. 15%) (Coef, 1.033; 95% CI, 0.833–1.233), followed by minimal sedation (vs. deep sedation) (Coef, 0.609; 95% CI, 0.448–0.769). In addition, the parents preferred lower costs and shorter fasting and recovery times (16%, 14%, and 10%, Figure 2).

**Table 3:** Preferences of parents for different features of procedural sedation in pediatric dentistry.

		95% CI		** **	** **
Attribute	Coefficient	LB	UB	SE	*p-*value
Fasting					
No requirement	0.426	0.275	0.578	0.077	**<0.001**
0 h for clear fluids	0.222	0.108	0.336	0.058	**<0.001**
3 h for milk and solids					
2 h for clear fluids	0	NA	NA	NA	NA
6 h for milk and solids	[Reference]				
Recovery time					
10 min	0.310	0.186	0.435	0.064	**<0.001**
30 min	0.194	0.076	0.311	0.060	**0.001**
90 min	0 [Reference]	NA	NA	NA	NA
Administration route of sedation drug					
Oral	0.069	−0.091	0.230	0.082	0.397
Inhalation	0.195	0.015	0.375	0.092	**0.033**
Intranasal	−0.037	−0.181	0.107	0.074	0.617
Intravenous	0 [Reference]	NA	NA	NA	NA
Adverse reaction incidence					
0 in 100 people (0%)	1.033	0.833	1.233	.1018	**<0.001**
5 in 100 people (5%)	0.581	0.424	0.738	0.080	**<0.001**
15 in 100 people (15%)	0 [Reference]	NA	NA	NA	NA
Sedation depth					
Minimal sedation	0.609	0.448	0.769	0.082	**<0.001**
Moderate sedation	0.514	0.359	0.669	0.079	**<0.001**
Deep sedation	0 [Reference]	NA	NA	NA	NA
Cost	−6.715 × 10^−4^	−9.467 × 10^−4^	−3.963 × 10^−4^	1.404 × 10^−4^	**<0.001**

LB, lower bound; UB, upper bound; NA, not applicable.

### 3.3. The impact of parental dental anxiety

Figure 2 shows the relative importance of each attribute in decision-making regarding procedural sedation among parents with or without dental anxiety. The relative importance of adverse reactions was higher among anxious parents than nonanxious parents (37% vs. 29%), as was the relative importance of fasting time (18% vs. 9%). Conversely, anxious parents placed far less emphasis on cost than nonanxious participants (7% vs. 25%). This result suggests that parents with dental anxiety valued the incidence of adverse effects and fasting time more and were willing to pay more for it. The relative importance of the recovery time, administration route of the sedation drug and the depth of sedation was relatively close among anxious and nonanxious parents (10% vs. 11%; 9% vs. 4%; 19% vs. 22%).

### 3.4. Willingness to Pay (WTP)

**Figure 3:**
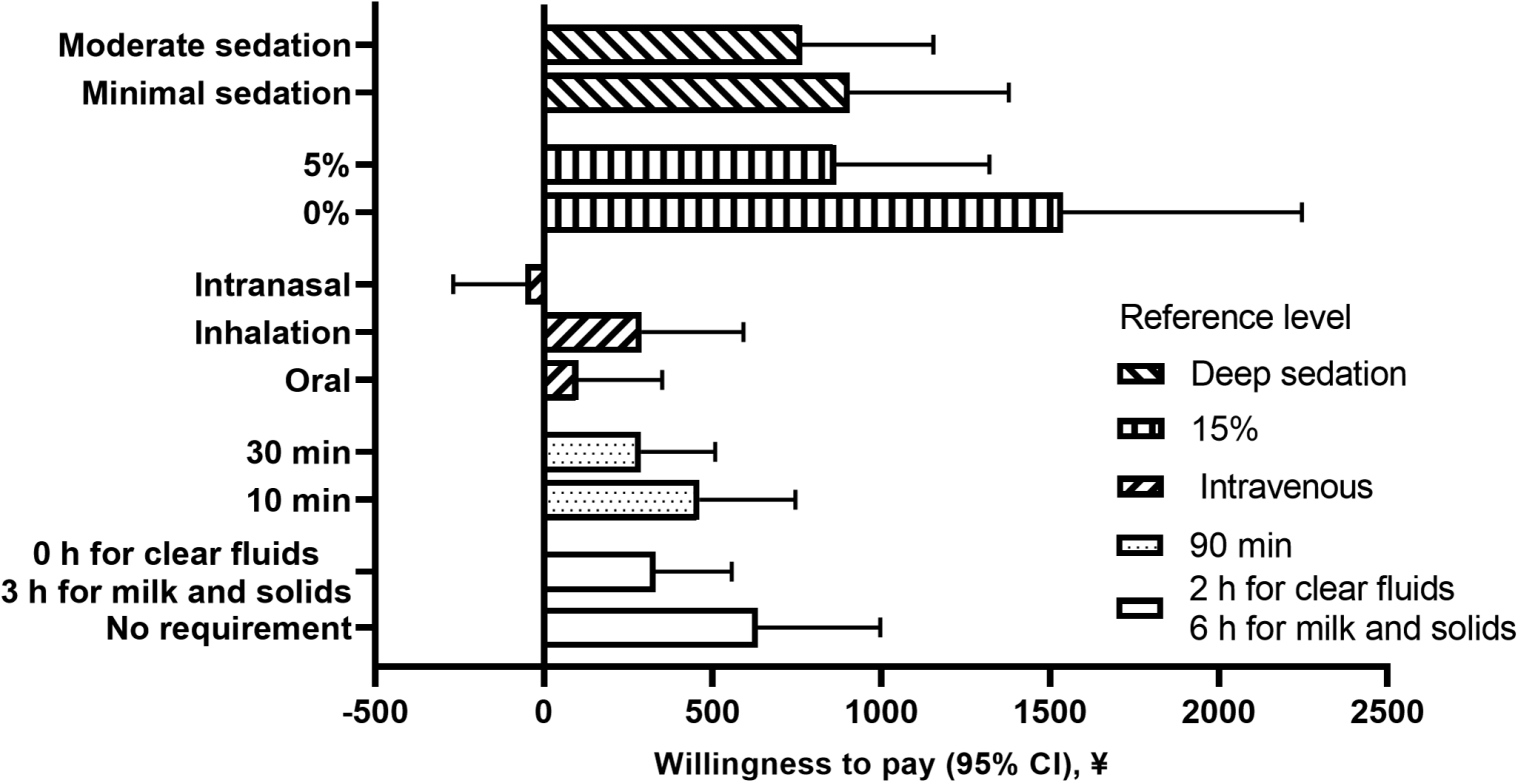
Willingness to pay for level changes in specific attributes.

Figure 3 shows the WTP threshold for level changes in specific attributes, representing the parents' trade-off of costs for sedation treatment. We defined a base procedure sedation choice set: 2 h for clear fluids, 6 h for milk and solids for fasting times, 90 min of recovery time, intravenous administration, 15% incidence of adverse reactions and deep sedation. The WTP threshold for a lower rate of adverse reactions [0% rate: ¥1,538 (95% CI, ¥829–¥2,247); 5% rate: ¥866 (95% CI, ¥412–¥1,320)] showed that patients were willing to pay more for procedural sedation options associated with fewer adverse reactions in their children. Parents' WTP threshold for minimal sedation was ¥906 (95% CI, ¥435–¥1,378), and ¥766 (95% CI, ¥376– ¥1,155) for moderate sedation. The parents were also willing to pay ¥634 (95% CI, ¥272–¥997) for sedation treatment without fasting. Additionally, they were prepared to pay ¥462 (95% CI, ¥179–¥746) for a 10 min recovery time and ¥288 (95% CI, ¥68–¥508) for a 30 min recovery time. Based on the WTP results, the WTP threshold for the best sedation scenario (no fasting requirement, 10 min recovery time, administration by inhalation, 0% adverse reaction incidence and minimal sedation) was ¥3,830 (95% CI, ¥1,704–¥5,960), amounting to approximately $545, (95% CI, $243–$851).

## 4. Discussion

This study is the first to quantify parents' benefit-risk preferences and their trade-offs regarding sedation treatment in pediatric dentistry using DCEs. Our study showed that parents of pediatric dental patients preferred a shorter fasting and recovery time, fewer adverse reactions, administration by inhalation, a milder depth of sedation and lower out-of-pocket cost. Parents had a greater WTP threshold for reducing the incidence of adverse reactions than for changing other attributes.

Our study shows that adverse reactions are parents' highest priority. The most significant adverse reactions to sedation in children are respiratory depression, followed by nausea and vomiting, circulatory depression and accidental aspiration (42). Numerous experiments assessing parents' treatment preferences for their children have revealed that parents value reducing adverse reactions (43–45). Adverse reactions were the most important attribute in a DCE assessing parents' preferences for new combination vaccines in their children (45). A study of parents' preferences regarding adolescent depression treatment also revealed a strong preference for treatments with lower adverse event incidences (43). In addition, relevant literature shows that reducing the adverse reactions of sedation increases parental satisfaction (42, 46, 47). Furthermore, the WTP analysis revealed that parents were willing to spend the most to reduce adverse reactions, emphasizing the importance parents place on reducing adverse reactions to sedation. Another DCE showed that emergency physicians attach great importance to the adverse effects of procedural sedation in children (23), which suggests that doctors were likely already aware of the parental preference and have begun to use this observation in clinical practice.

Second, our findings show that parents choosing procedural sedation in pediatric dentistry prefer minimal sedation and are willing to pay ¥906 (approximately $129) for it. Minimal and moderate sedation techniques are widely used in pediatric dentistry (48). While minimal and moderate sedation can be unpredictable compared to deep sedation, especially in complex and invasive dental procedures (48), using a higher depth of sedation can increase the chance of adverse events (49). In the current era of individualized medicine, there is a trade-off between the need for deep sedation and parental preference in pediatric dentistry, presenting a challenge for physicians in clinical practice. Fortunately, studies have revealed that the sedative dose can be reduced when patients listen to music or nature sounds of nature (50, 51), which may be a potential future development opportunity.

Because of concerns about pulmonary aspiration, guidelines commonly recommend a minimum period of fasting prior to elective sedation: 2 h or longer for clear liquids, 4 h or longer for breast milk, and 6 h or longer prior for cow milk, infant formula, or a light meal (52–54). However, fasting before procedural sedation has minimal scientific support; thus, current fasting recommendations from prominent specialty societies are largely consensus driven (55). Some studies have found no apparent association between aspiration and noncompliance with fasting recommendations in children (56, 57). In addition, fasting is regularly incomplete before procedural sedation in other settings, such as coronary interventions, oral contrast (58) and eye surgery (59), and no increased aspiration risk has been shown in the above settings. Given the low observed frequency of aspiration, fasting strategies in procedural sedation can reasonably be less restrictive (55). In our study, parents preferred shorter fasting times and were willing to spend ¥634 (approximately $90) to prevent their children from having to fast. Therefore, we must encourage doctors to choose a reasonable fasting time in their daily practice, depending on the required dental procedures and parental preference.

Regarding other attributes of procedural sedation, our study shows that parents preferred shorter recovery times, inhalation sedation and lower cost. Another study found that parents prefer shorter recovery times (60), which is consistent with our experimental results. When sedatives are administered, parents prefer inhalation sedation, which should arouse the attention of doctors and researchers and promote relevant clinical applications and experimental research. Inhaled nitrous oxide is now widely used for clinical dental sedation, but its effects on children are sometimes unpredictable (42). One study showed that when the aerosolized form of midazolam was compared to the drop form, the aerosolized form was more well tolerated and produced less adverse behavior, and the effect was more stable than intranasal midazolam (61). Overall, continuous studies are needed to improve the sedative administration routes and recovery time of procedural sedation.

This study is the first to examine the effect of dental anxiety on parental preferences for pediatric dental sedation treatment. Adverse reactions were the highest priority regarding children's procedural sedation among the total respondents, and parents with dental anxiety had a stronger preference for a lower incidence of adverse reactions than those without dental anxiety. Second, parents with dental anxiety prioritized shorter fasting times more than those without dental anxiety. The child's behavioral response to dental treatments may be linked to parental dental anxiety (62). One study revealed that parental dental anxiety was associated with the avoidance of dental treatment in children (63). A discrete choice trial of emergency physician preferences for procedural sedation fasting in children revealed that extreme parental concern for children leads doctors to choose longer fasting times (23). This choice is in contrast with the needs of parents with dental anxiety according to our study, as we found that parents prefer procedural sedation options with a shorter fasting time. Although sedation depth is an important attribute in pediatric dental sedation, there was not much difference between dental anxious parents and nondental anxious patients in the importance of this attribute. One study showed that parental dental anxiety does not influence parents' choice between sedation and general anesthesia for their children (64), and the results of our study may explain this finding in terms of parental preferences. In addition, although parents preferred procedural sedation with lower out-of-pocket expenses, parents with anxiety paid less attention to the cost of sedation, indicating that they were willing to spend more money on procedural sedation for their children.

This study has some limitations. First, because it was not possible to include all attributes of procedural sedation in the selection set, only six of the most representative attributes were selected in this study through a qualitative research approach. Therefore, the DCEs may not represent all complex procedural sedation choices given the limited number of attributes and levels. Second, although the 186 respondents provided adequate power for this study, the relatively small sample size suggests that our findings may not be generalizable to China as a whole. Future studies should increase the sample size accordingly. Furthermore, with larger sample sizes, the influence of other demographic characteristics on preferences should be investigated further.

In addition to these points, there are some questions that need to be addressed in future studies: How do the specific dental procedures and their associated complexities influence parental preferences for sedation attributes, and are there any differences in preferences between routine and more invasive procedures? What are the most effective strategies for addressing parental anxiety and enhancing communication between healthcare providers and parents to optimize the decision-making process for procedural sedation? Addressing these research questions in future studies will contribute to a more comprehensive understanding of parental preferences for pediatric procedural sedation and improve overall dental care experiences for children and their families.

## 5. Conclusion

This study demonstrates that DCEs are a valid and accurate preference survey method that quantify different treatment attributes and their levels to provide useful information to help clinicians/researchers understand the relative importance of parental preferences for sedation treatment in pediatric dentistry. Parents of children undergoing dental treatments prefer an option with a lower incidence of adverse reactions, followed by a lower sedation depth, shorter fasting and recovery time and inhalation sedation. Our results also showed that parents with dental anxiety had a stronger preference for an option with a lower incidence of adverse reactions and a shorter fasting time than parents without dental anxiety. Understanding parental treatment preferences through DCEs and taking them into account in clinical decision-making can facilitate decision-sharing between patients and physicians, which in turn can improve the compliance of children and their parents, reduce pediatric dental anxiety, and assist in smoother pediatric dental treatment.

## Data Availability

The raw data supporting the conclusions of this article will be made available by the authors, without undue reservation.
